# O-17 - 1 UNDERSTANDING AND PREVENTING HARMS CAUSED BY CLIMATE CHANGE RISKS IN PRISONS IN WALES

**DOI:** 10.1093/pubmed/fdag046.015

**Published:** 2026-07-09

**Authors:** Mr Adam Davies, Dr Benjamin Gray, Dr Aimée Challenger, Dr Stephanie Perrett, Mr Kristian James

**Affiliations:** Public Health Wales; HM Prison and Probation Service in Wales; Public Health Wales; Public Health Wales; Public Health Wales; Public Health Wales

## Abstract

**Background:**

Risks to prison settings from climate change (CC) have been identified through UK and Wales risk assessment (CCRA) processes. Published evidence of health risks of CC to prisoners and staff is limited. Risks include heat related deaths, flooding, severe weather damaging buildings, power outages, and risks to food and water supply. Whilst CC is driven by global warming, a lack of heating remains a recognised problem. Adaptation to risks may be required, but this is also presently unknown.

**Objectives:**

To assist prisons, identify CC risks to inform adaptation planning and preparedness.

**Methods:**

A collaboration between Public Health Wales (PHW), His Majesty’s Prison and Probation Service (HMPPS) in Wales and the Ministry of Justice was established. To identify CC risks, PHW developed a checklist based on existing sector guidance and CCRA evidence. The questionnaire was administered by HMPPS. Initially trialled at a single prison with feedback incorporated before rollout. The checklist could be completed by prison and estate managers, health and safety and medical staff.

**Results:**

All six prisons completed the questionnaire. Each prison identified its own risks and level of adaptation. Some of these risks included inconsistencies around sunscreen provision across prison sites. Staff voiced concerns about a lack of summer uniform appropriate for hotter days. Opportunities for further activity included refreshing business continuity plans (to account for CC), considering the risks posed by wildfires at some sites and how to improve in-cell ventilation.

**Conclusions:**

The questionnaire has enabled each prison to consider impacts of CC hazards and state of adaptation. Without undertaking this checklist, the preparedness would have remained unknown to the sites. Repeating this process on a semi-regular basis to review climate preparedness and identify further mitigation measures is recommended. This checklist and approach could be adapted for other sectors e.g. care settings, schools.

